# Overweight, obesity, steps, and moderate to vigorous physical activity in children

**DOI:** 10.1590/S1518-8787.2017051006771

**Published:** 2017-04-18

**Authors:** Luis Carlos Oliveira, Gerson Luis de Moraes Ferrari, Timóteo Leandro Araújo, Victor Matsudo

**Affiliations:** I Centro de Estudos do Laboratório de Aptidão Física de São Caetano do Sul. São Caetano do Sul, SP, Brasil; II Universidade São Judas Tadeu. São Paulo, SP, Brasil; III Centro de Atendimento e Apoio ao Adolescente. Departamento de Pediatria. Universidade Federal de São Paulo. Escola Paulista de Medicina. São Paulo, SP, Brasil

**Keywords:** Child, Walking, classification, Motor Activity, Overweight, prevention & control, Pediatric Overweight, prevention & control

## Abstract

**OBJECTIVE:**

The objective of this study is to establish cutoff points for the number of steps/day and minutes/day of moderate to vigorous physical activity in relation to the risk of childhood overweight and obesity and their respective associations. In addition, we aim to identify the amount of steps/day needed to achieve the recommendation of moderate to vigorous physical activity in children from São Caetano do Sul.

**METHODS:**

In total, 494 children have used an accelerometer to monitor steps/day and the intensity of physical activity (min/day). The moderate to vigorous physical activity has been categorized according to the public health recommendation (≤ 60 *versus* > 60 min/day). Overweight or obesity is defined as body mass index > +1 SD, based on reference data from the World Health Organization. The data on family income, education of parents, screen time, diet pattern, and sedentary time have been collected by questionnaires. Logistic regression and Receiver Operating Characteristic curves have been constructed.

**RESULTS:**

On average, boys walked more steps/day (1,850) and performed more min/day of moderate to vigorous physical activity (23.1) than girls. Overall, 51.4% of the children have been classified as eutrophic and 48.6% as overweight or obese. Eutrophic boys walked 1,525 steps/day and performed 18.6 minutes/day more of moderate to vigorous physical activity than those with overweight/obesity (p < 0.05). The same has not been found in girls (p > 0.05). The cutoff points to prevent overweight and obesity in boys and girls were 10,500 and 8,500 steps/day and 66 and 46 min/day of moderate to vigorous physical activity, respectively. The walking of 9,700 steps/day for boys and 9,400 steps/day for girls ensures the scope of the recommendation of moderate to vigorous physical activity.

**CONCLUSIONS:**

In boys, steps/day and moderate to vigorous physical activity have been negatively associated with body mass index, regardless of race, family income, education of parents, screen time, diet pattern, and sedentary time. We suggest, for steps/day and moderate to vigorous physical activity, studies with different ages and populations, with different designs, so as to inform the cause and effect relationship with various health parameters.

## RESUMO

**OBJETIVO:**

Estabelecer pontos de corte para o número de passos/dia e min/dia de atividade física com intensidade moderada a vigorosa em relação ao risco do excesso de peso e obesidade infantil e suas respectivas associações. Além de identificar a quantidade de passos/dia necessários para atingir a recomendação de atividade física de moderada a vigorosa em crianças de São Caetano do Sul.

**MÉTODOS:**

No total, 494 crianças usaram acelerômetro para monitorar os passos/dia e a intensidade da atividade física (min/dia). A atividade física de moderada a vigorosa foi categorizada de acordo com a recomendação de saúde pública (≤ 60 *versus* > 60 min/dia). Excesso de peso ou obesidade foi definido como índice de massa corporal > +1 DP, com base nos dados de referência da Organização Mundial de Saúde. Renda familiar, escolaridade dos pais, tempo de tela, padrão de dieta, e tempo sedentário foram coletados por questionários. Foram construídas curvas *Receiver Operating Characteristic* e regressão logística.

**RESULTADOS:**

Em média, os meninos realizaram mais passos/dia (1.850) e min/dia de atividade física de moderada a vigorosa (23,1) do que as meninas. No geral, 51,4% das crianças foram classificadas como eutróficas e 48,6% com excesso de peso ou obesidade. Meninos eutróficos realizavam 1.525 passos/dia e 18,6 min/dia de atividade física de moderada a vigorosa a mais do que aqueles com excesso de peso/obesidade (p < 0,05). O mesmo não foi encontrado nas meninas (p > 0,05). Os pontos de corte para evitar excesso de peso e obesidade nos meninos e meninas foram 10.500 e 8.500 passos/dia e 66 e 46 min/dia de atividade física de moderada a vigorosa, respectivamente. A realização de 9.700 passos/dia (meninos) e 9.400 passos/dia (meninas) garante o alcance da recomendação de atividade física de moderada a vigorosa.

**CONCLUSÕES:**

Nos meninos, passos/dia e atividade física de moderada a vigorosa foram negativamente associados com o índice de massa corporal, independentemente da raça, renda familiar, escolaridade dos pais, tempo de tela, padrão de dieta e tempo sedentário. Sugere-se para passos/dia e atividade física de moderada a vigorosa, estudos em diversas idades e populações, com diferentes delineamentos, para assim informar a relação causa e efeito com diversos parâmetros de saúde.

## INTRODUCTION

The increasing prevalence of overweight and obesity in children around the world is a critical public health problem that has encouraged Governments to consider different strategies to reduce obesity in the population^1^. The National Survey of School Health (2010) has reported that 33.5% of Brazilian children were overweight, and 16.6% of boys and 11.8% of the girls were obese^2^.

The beneficial effect of regular physical activity on the health of children is well established^3^ ^,^ ^4^. Increased physical activity has been associated with improvements in cardiovascular risk factors^4^. Therefore, the objective measurement of physical activity using accelerometers is important, both for surveillance purposes and in the evaluation of the effectiveness of public health interventions, because it provides detailed data, such as the number of steps/day and moderate to vigorous physical activity (MVPA). The use of accelerometers has become more common in research studies on physical activity in childhood in high-income countries^3^ ^,^ ^5^. On the other hand, there are relatively few studies that have used this technology in low-and middle-income countries, such as Brazil^6^.

A cumulative record of steps and minutes performed throughout the day are adequate markers to control the accumulation of daily physical activity in youth, as it has been the focus of public health guidelines^3^ ^,^ ^7^ ^,^ ^8^. The recommendations establish that children and adolescents of both genders should walk at least 12,000 steps/day^9^ or accumulate at least 60 min/day of MVPA^7^. In addition, Tudor-Locke et al.^10^ have proposed different values for boys (15,000 steps/day) and girls (12,000 steps/day) in order to prevent childhood overweight and obesity, measured by body mass index (BMI).

Evidence shows negative association between steps/day and MVPA (objectively measured) and adiposity^6^ ^,^ ^11^ ^,^ ^12^. Several research studies^6^ ^,^ ^12^ ^,^ ^13^ have found negative relationship between steps/day and BMI and body fat percentage (%BF). Basterfield et al.^12^ have reported greater time spent in MVPA in boys than in girls (28 *versus* 21 min/day). In addition, the authors have found associations between MVPA and fat mass index and BMI in boys; however, in girls, the authors have found no association. Jimenez-Pavon et al., in a systematic review, have concluded that MVPA was associated with variables of body composition more consistently in boys than in girls^11^.

Nevertheless, the question about how many steps/day and min/day of MVPA are required to prevent overweight and obesity in children of low- and middle-income countries remains to be elucidated, since research studies that use objective instruments to measure physical activity in Brazilian children are still scarce. In a literature review, Tudor-Locke et al.^8^ have found no Brazilian study that has used objective methods to quantify the steps/day necessary to attain the MVPA guidelines^7^. Therefore, the objectives of this study were: 1) to establish cutoff points for the number of steps/day and min/day of MVPA in relation to the risk of overweight and obesity and their respective associations, and 2) to identify the amount of steps/day needed to achieve the recommendation of MVPA^7^ in children from São Caetano do Sul.

## METHODS

This study is part of the International Study of Childhood Obesity, Lifestyle and the Environment (ISCOLE) carried out in twelve countries, involving the five major geographical regions of the world. Details of the protocol and sampling calculation of the ISCOLE are described in another publication^5^.

This study focuses on data collected by ISCOLE Brazil, carried out in the city of São Caetano do Sul, State of São Paulo, Brazil, with a territorial area of 15.3 km^2^. In 2013, the population of the municipality aged 10 years consisted of 1,557 children (812 boys)^14^. Initially, the Municipal Education Department was contacted and, upon approval, the project was implemented in schools, and children in the fifth grade of the elementary school were invited to be part of the study.

Data collection took place between March 2012 and April 2013 and all evaluations were performed during one full week per school. All activities of data collection and management were carried out and monitored by the coordinating center of ISCOLE^5^.

For the selection of schools, we generated random lists of public and private schools of the municipality, and we selected schools from each list in the proportion of four public schools to one private school. In the case of refusal to participate in the project by the school, it was replaced by the following school in the list. In total, sixteen public and four particular schools participated in the research, with a sample of 25-30 children per school.

In total, 564 children (277 boys) were evaluated and met the following inclusion criteria: (a) being aged between nine and eleven years, (b) being regularly enrolled in a school of the municipality, and (c) having no clinical or functional conditions that limited the practice of daily physical activity. The exclusion criteria adopted were: invalid data of the accelerometry and anthropometry. The parents or guardians signed the informed consent and the study was approved by the Ethics Committee of the Universidade Federal de São Paulo (Process 332,529, July 12, 2013).

The accelerometer Actigraph GT3X was used to monitor objectively the steps/day, the MVPA, and the sedentary time. The accelerometer was used in the waist in an elastic belt, in the axillary line on the right side. Participants were encouraged to use the accelerometer 24 hours/day for at least seven days (plus one day of initial familiarization and on the morning of the last day), including two days in the weekend. The minimum amount of accelerometer data considered as acceptable was four days (including at least one weekend day), with at least 10 hours/day of use, after removing for sleep^15^ ^,^ ^16^. Blocks of 20 consecutive minutes with zero count were considered as non-use of the equipment and removed from the analyses.

After the last day of data collection, the team went to the school to remove the accelerometers. We used the software Actilife, version 5.6. Nine participants who did not provide sufficient data for the initial monitoring used the accelerometer in the second week to ensure that the minimum data requirements were met. The data were collected at a sampling rate of 80 Hz, downloaded in periods of one second, and aggregated for periods of 15 seconds^17^.

We used counts for the cutoff points of accelerometers established by Evenson et al.^17^ for periods of 15 seconds. We use the cutoff point of ≤ 25 counts/15 seconds for sedentary time and ≥ 574 counts/15 seconds for MVPA^17^.

Height, body weight, and BMI were measured according to previously standardized procedures^5^. Height was measured with the children without shoes using a portable stadiometer, Seca 213, with the head facing the Frankfurt plane. Body mass was measured using a scale, Tanita SC-240, portable analyzer of body composition, after removing heavy items from pockets, shoes, and socks^18^. Two measurements were obtained, and the average was used for analysis. If the first two measurements had a difference greater than 0.5 kg or 2% of body mass, a third measurement was carried out.

The BMI was obtained by dividing body weight in kilograms (kg) by height in meters squared (m^2^). The BMI of the children from the study was compared to the BMI of growth curves of the World Health Organization (WHO) made for children and adolescents according to age and gender, and converted into standard deviation (SD) of the median^19^. With this, the values proposed by the WHO were used as classification criteria and participants were classified into eutrophic (< +1 SD) and overweight or obese (≥ +1 SD)^19^.

A parent or legal guardian was invited to fill in the Neighborhood and Home Environment Questionnaire, which included questions related to the health history of the child, annual household income, and the educational level of the parents^5^. Annual family income (R$) was classified into four categories that represent increasing levels of income. The level of combined education of the parents (highest level of any of the parents) was used from the combination of both responses of the parents.

The Diet and Lifestyle Questionnaire was used to collect data related to food consumption, sedentary behaviors, and screen time of the children^5^. The children completed the questionnaire related to the consumption of 23 food items in a regular week. To identify existing food patterns, we conducted a principal component analysis (PCA), considering the food items as input variables. The PCA was performed with the varimax orthogonal transformation to force the non-correlation and to improve interpretation. Two factors were identified: unhealthy diet pattern (fast food, French fries, ice cream, pastries, pies, sweets, among others) and standard healthy diet (vegetables, orange, fruit juice, fruits, among others). Both scores were considered separately and addressed as continuous variables. The highest values for each score represent an unhealthy or healthy diet pattern, respectively.

The children were asked how many hours they usually watch TV, play video games, or use the computer during the week and in the weekend^5^. Total screen time was calculated by adding the time for TV and video or computer games.

We performed a descriptive analysis, and the Kolmogorov-Smirnov test was used to evaluate the distribution of the data. We used Student’s t-test for independent samples and chi-square test for categorical variables.

The predictive power and the cutoff points of steps/day and MVPA (min/day) for the prevention of overweight and obesity were identified using the Receiver Operating Characteristic (ROC) curves. Initially, we identified the total area under the ROC curve between the number of steps/day, MVPA (min/day), and the prevention of overweight and obesity. The bigger the area under the ROC curve the greater the discriminatory power, and we also used a 95% confidence interval (95%CI). The calculation of the 95%CI determines whether the predictive capability is not due to chance, and its limit must be greater than 0.50^20^. Then, we calculated sensitivity and specificity, as well as cutoff points for the number of steps/day and MVPA for the prevention of overweight and obesity. We also present the areas under the ROC curve for the identification of the steps/day needed to achieve the recommendation of MVPA (≤ 60 *versus* > 60 min/day)^7^.

The cutoff points found for the number of steps/day and MVPA (min/day) were used to create new dichotomous variables (below and above the cutoff point). These variables were considered as independent variables in logistic regression models, to quantify the effect that the number of steps and minutes of MVPA above the cutoff points have on the risk of overweight and obesity. Models were made separated by gender, unadjusted and adjusted for race, income, education of parents, screen time, unhealthy and healthy diet, and sedentary time. The effects of the regression models were evaluated by odds ratio (OR) and their 95%CI. We used the Statistical Package for the Social Sciences (SPSS, version 22.0) for the analyses, considering a significance level of 5%^21^.

## RESULTS

The sample consisted of 494 children (242 boys). There was no difference between the genders for average age and race. On average, boys used the accelerometer 14 min/day more than girls (p < 0.05). However, we found no significant differences between the genders in the number of days of use of the accelerometer. For the total number of steps/day and MVPA, the boys walked on average 1,850 steps/day and performed 23.1 min/day more of MVPA than girls (p < 0.05). Regarding sedentary time, boys had on average 15.2 min/day more than girls (Table 1).

**Table 1:** Descriptive analysis of accelerometry, anthropometry, family income, education of parents, screen time, and diet pattern of children from São Caetano do Sul, State of São Paulo, Brazil.

Variable	Boys	Girls	p
	
	(n = 242)	(n = 252)	
Age (years) – average (SD)	10.2 (0.6)	10.1 (0.5)	0.961^a^
Race – n (%)			0.292^b^
White	158 (71.8)	172 (74.8)	
Black	15 (6.8)	20 (8.7)	
Brown	40 (18.2)	28 (12.2)	
Other	7 (3.2)	10 (4.3)	
Accelerometry – average (SD)			
Time of use of the accelerometer (min/day)	905 (51)	891 (50)	**< 0.001** ^a^
Number of days	6.9 (1.1)	6.8 (1.2)	0.074^a^
Steps/day	10,570 (2,915)	8,720 (2,258)	**0.004** ^a^
MVPA (min/day)	71.33 (28.02)	48.23 (18.08)	**< 0.001** ^a^
Sedentary time (min/day)	492.18 (70.15)	507.35 (66.70)	**0.014** ^a^
Anthropometry – average (SD)			
Body mass (kg)	41.5 (12.6)	41.1 (10.9)	**0.014** ^a^
Height (dm)	144.5 (7.2)	145.3 (7.7)	0.130^a^
BMI (kg/m^2^)	19.9 (4.7)	19.5 (4.1)	**0.017** ^a^
Categories of BMI (cutoff point WHO)^19^ – n (%)			0.537^b^
Eutrophic	121 (50.0)	133 (52.8)	
Overweight/Obesity	121 (50.0)	119 (47.2)	
Annual family income – n (%)			0.070^b^
≤ R$19,620.00	74 (37.4)	76 (39.4)	
R$19,620.01 to R$32,700.00	63 (31.8)	40 (20.7)	
R$32,700.01 to R$58,860.00	38 (19.2)	46 (23.8)	
≥ R$58,860.01	23 (11.6)	31 (16.1)	
Education level of the parents – n (%)			0.648^b^
Incomplete high school	57 (25.9)	51 (22.2)	
Complete high school or college	115 (52.3)	127 (55.2)	
Specialization	48 (21.8)	52 (22.6)	
Screen time (hours/day) – average (SD)			
Total screen time	4.14 (2.22)	3.70 (2.20)	**0.026** ^a^
TV	2.35 (1.42)	2.31 (1.35)	0.728^a^
Computer/Video games	1.80 (1.37)	1.39 (1.29)	**0.001** ^a^
Unhealthy diet	3.16 (1.00)	2.86 (0.77)	**< 0.001** ^a^
Healthy diet	3.88 (1.21)	3.77 (1.10)	0.280^a^

MVPA: moderate to vigorous physical activity; BMI: body mass index; WHO: World Health Organization; TV: television

Values with statistical significance presented in bold.

^a^ Value of significance of Student’s t-test for independent samples.

^b^ Value of significance of the Chi-square test.

We found significant differences in average body mass and BMI according to gender: boys showed higher values than the girls. Almost half (48.6%) of the children were overweight or obese. As for height, no significant differences were observed (Table 1).

Most of the families had annual income of less than R$19,620.00 (38.4%) and parents who had completed high school or college (53.8%). For these variables, no significant differences were found (Table 1).

On average, boys had more total screen time (26.4 min/day) and video/computer games (24.6 min/day) than girls. No difference was found between genders for TV time. The unhealthy diet pattern of boys was, on average, statistically higher than the pattern of girls. The same did not occur for the healthy diet pattern (Table 1).

On average, boys classified as eutrophic walked 1,525 steps/day and performed 18.6 minutes/day of MVPA more than boys with overweight or obesity. Among the girls, the eutrophic ones walked, on average, 500 steps/day and performed 3.82 minutes/day of MVPA more than girls with overweight or obesity, but the differences were not significant (Table 2).

**Table 2:** Comparison (n, average, and SD) of the number of steps/day and moderate to vigorous physical activity (min/day) according to school children classified as eutrophic and overweight/obese.

Variable	Eutrophic			Overweight/Obese			p*
		
Steps/day	n	Average	SD	n	Average	SD	
Boys	121	11,539.94	3026.06	133	9623.71	2463.39	**< 0.001**
Girls	121	8925.71	2191.31	119	8491.28	2319.64	0.128
MVPA (min/day)							
Boys	121	80.62	28.33	133	62.05	24.49	**< 0.001**
Girls	121	50.03	18.15	119	46.21	17.86	0.094

MVPA: moderate to vigorous physical activity

Values with statistical significance presented in bold.

* Value of significance of Student’s t-test for independent samples.

In boys, the area under the ROC curve shows that the number of steps has good capacity to discriminate eutrophic boys from those with overweight or obesity. The cutoff point for steps/day found (10,502) ensures a sensitivity of 66.7% and a specificity of 64.8%. In girls, the discriminatory capacity was lower and the cutoff point found (8,540) indicates low values of sensitivity (58.1%) and specificity (55.1%).

The MVPA (min/day) had better ability to discriminate boys classified as eutrophic from those with overweight or obesity with a cutoff point of 66.70 min/day, showing sensitivity of 65.3% and specificity of 63.6%. In girls, the ability to discriminate girls classified as eutrophic from those with overweight or obesity was smaller, with cutoff of 46.59 min/day, sensitivity of 55.6%, and specificity of 54.6%, respectively.

**Figure 1:**
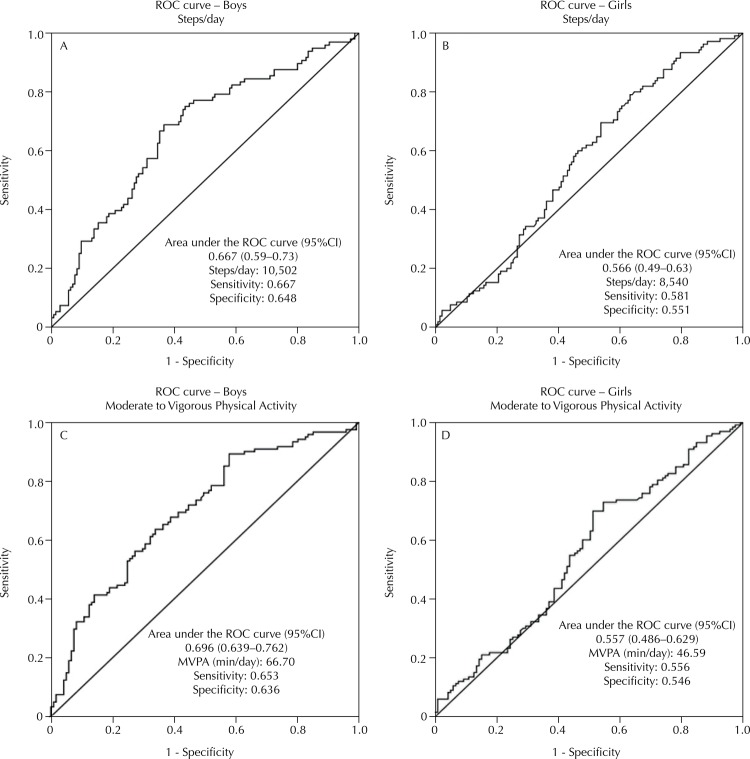
Area under the ROC curve, 95%CI, cutoff points, sensitivity, and specificity for overweight/obesity from the number of steps/day (A: boys; B: girls) and moderate to vigorous physical activity (min/day; C: boys; D: girls) of children. São Caetano do Sul, State of São Paulo, Brazil.

Table 3 shows the results of the logistic regression models, with the dichotomous variables as the independent variables from the cutoff points of Figure 1, and the BMI as the dependent variable. Only in boys the number of steps/day and the min/day of MVPA had significant effect on the classification of BMI.

**Table 3:** Logistic regression models for the study of the influence of the number of steps/day and moderate to vigorous physical activity (min/day) in overweight/obesity (dependent variable: 0 = eutrophic, 1 = overweight or obese).

Variable	Unadjusted model			Adjusted model^a^		
	
	β	p	OR (95%CI)	β	p	OR (95%CI)
Boys						
Steps/day^b^	-1.452	**< 0.001**	0.234 (0.137–0.401)	-1.575	**< 0.001**	0.207 (0.103–0.414)
Girls						
Steps/day^c^	-0.446	0.079	0.640 (0.389–1.053)	-0.347	0.319	0.707 (0.358–1.397)
Boys						
MVPA (min/day)^d^	-1.191	**< 0.001**	0.304 (0.179–0.514)	-1.114	**0.001**	0.328 (0.170–0.635)
Girls						
MVPA (min/day)^e^	-0.412	0.104	0.662 (0.403–1.089)	-0.403	0.238	0.668 (0.342–1.304)

MVPA: moderate to vigorous physical activity

Values with statistical significance presented in bold.

^a^ Adjusted for race, annual household income, education of parents, screen time, unhealthy diet pattern, healthy diet pattern, sedentary time.

^b^ Dichotomous variable: 0 = ≤ 10502; 1 = > 10502

^c^ Dichotomous variable: 0 = ≤ 8540; 1 = > 8540

^d^ Dichotomous variable: 0 = ≤ 66.70; 1 = > 66.70

^e^ Dichotomous variable: 0 = ≤ 46.59; 1 = > 46.59

MVPA: moderate to vigorous physical activity

Boys with more than 10,502 steps/day were 79.3% less likely to be classified as overweight or obese, regardless of race, income, education of parents, screen time, diet pattern, and sedentary time. As for MVPA (min/day), considering the adjusted effect, boys were 67.2% less likely to be classified as overweight or obese (Table 3).

**Figure 2:**
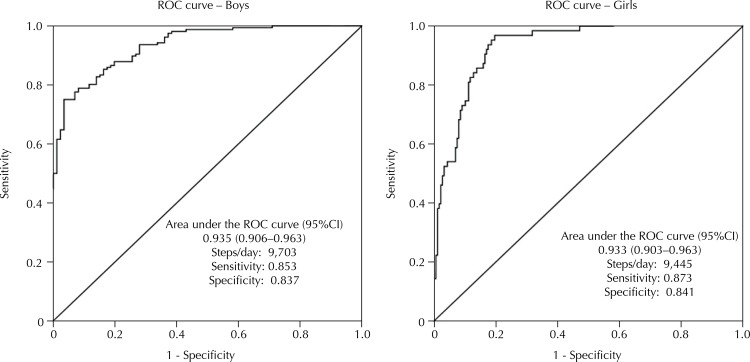
Area under the ROC curve, 95%CI, cutoff points, sensitivity, and specificity of the number of steps/day as the discriminator for the recommendation of MVPA (≤ 60 *versus* > 60 min/day)7 of children. São Caetano do Sul, State of São Paulo, Brazil.

In Figure 2, we present the areas under the ROC curve for the identification of the number of steps/day needed to achieve the recommendation of 60 min/day of MVPA^7^. In both genders, the amount of steps/day has an excellent ability to discriminate those who comply from those who do not comply with the recommendation of MVPA^7^. In the case of boys, 9,703 steps/day ensures over 60 min/day of MVPA. In girls, 9,445 steps/day ensures 60 min/day of MVPA.

MVPA: moderate to vigorous physical activity

## DISCUSSION

Based on the objectives proposed, we could establish cutoff points for the number of steps/day and min/day of MVPA in relation to the risk of overweight and obesity and their respective associations, as well identify the amount of steps/day needed to achieve the recommendation of MVPA^7^ in children from São Caetano do Sul. The results showed a good discriminatory power for the number of steps/day and MVPA for the prevention of overweight/obesity in boys. Boys who reached the cutoff points (10,502 steps/day and 66.70 min/day of MVPA) had lower risks of being classified as overweight/obese. In girls, the numbers of steps/day and MVPA revealed low discriminatory power, leading to the identification of a cutoff point (8,540 steps/day and 46.59 min/day) with low sensitivity and specificity. In addition, we show a negative association between steps/day and MVPA with overweight/obesity in boys. In both genders, the amount of steps/day has an excellent ability to discriminate those who comply from those who do not comply with the recommendation of MVPA^8^. In the case of boys, we found a cutoff point of 9,703 steps/day and for girls 9,445 steps/day.

The relevance of these results in the promotion of the physical activity and health of children is justified by several factors, such as the well-known effect of physical activity on cardiovascular risk of children^4^ and specifically on childhood obesity^13^. In a cross-sectional study with children from Greece and using pedometers to measure steps/day, Michalopoulou et al.^13^ have found that eutrophic children of both genders walked more steps per day than those overweight^13^.

The cutoff points identified in this study for steps/day are below those proposed recently in other populations^10^. In addition to BMI, the method used in this study, %BF has also been used to establish the cutoff point of steps/day. Duncan et al.^22^ have analyzed children from New Zealand, Polynesia, and Asia using %BF to classify children with overweight and they have found greater cutoff point values (16,000 steps/day for boys and 13,000 for girls) than our study. Tudor-Locke et al.^10^ and Duncan et al.^22^ have used pedometers to quantify the cutoff point of steps/day to prevent overweight and obesity. Despite the difference between pedometers and accelerometers, both have high values of concordance and validation^23^ to measure steps/day. Pedometers are inexpensive, reliable, and easy to use and interpret, providing only the amount of steps/day^8^ ^,^ ^10^. On the other hand, accelerometers provide different intensities of physical activity and sedentary time^3^ ^,^ ^6^ ^,^ ^15^.

The average of steps/day in children from other countries^10^ (15,118 and 12,552 steps/day in eutrophic and overweight boys, respectively; 12,290 and 11,217 steps/day in eutrophic and overweight girls, respectively) was superior to that found in our study (11,539 and 9,623 steps/day in eutrophic and overweight boys, respectively; and 8,925 and 8,491 steps/day in eutrophic and overweight girls, respectively). However, both studies have shown that eutrophic children of both genders walked, on average, more steps/day than those who were overweight. Negative association between MVPA and BMI has also been found previously by Ferrari et al.^6^

Regarding the comparison of steps/day and MVPA between boys and girls, our results (10,570 *versus* 8,720 steps/day; 71.33 *versus* 48.23 min/day) corroborate several studies^6^ ^,^ ^10^ ^,^ ^13^ in which boys walked more steps/day and performed more MVPA than girls. Michalopoulou et al.^13^ have used pedometers to evaluate steps/day and have noticed that boys walked on average more steps/day than girls (14,069 *versus* 11,536 steps/day)^13^.

Based on our findings, boys and girls should do at least 67.69 and 45.59 of MVPA (min/day) to have lower risks of overweight and obesity. The results of this study are in accordance with the findings of research studies^6^ ^,^ ^24^ performed previously regarding the negative association of steps/day and MVPA with anthropometry in boys. In girls, similar results were not found. Stabelini Neto et al.^24^, using accelerometers, recommend that Brazilian children should do 88 min/day of MVPA to avoid the risk of metabolic syndrome.

Physical activity guidelines are expressed in frequency, time, and intensity^7^. No published information can be found with Brazilian children concerning the amount of steps/day needed to achieve the recommendation of MVPA. In addition, the identification of the amount of steps/day walked in daily life helps in the recommendation of the amount of moderate physical activity, also included in the physical activity guidelines^7^ ^,^ ^8^. To represent a total amount of 60 min/day of MVPA, Rowlands and Eston^25^ have used accelerometers to evaluate the MVPA of children from the United Kingdom and they have arrived to 13,000 steps/day for boys and 12,000 steps/day for girls.

This study expands the existing literature concerning the amount of steps/day, min/day of MVPA, and overweight and obesity in children aged between nine and eleven years. The study has some strong points: objective measure of steps/day and MVPA by accelerometer, a modern method that evaluates physical activity and which requires technological knowledge, a rare approach in Brazil, since most studies use a questionnaire to evaluate physical activity; and control, in the analysis, of the effect of race, annual household income, education level of parents, screen time, and diet of the children. On the other hand, this study also presents some limitations: a) as it is cross-sectional, the cause and effect relationship is limited. We have also not ruled out a possible reverse causality in which children who are not overweight or obese may have more willingness and ability to have a greater amount of steps/day and min/day of MVPA above the proposed values in the study than those overweight or obese; b) the non-representativeness of the sample prevents the extrapolation of the data to Brazilian children and even to the municipality of São Caetano do Sul in itself. In addition, São Caetano do Sul has a high human development index (0.86)^26^ in relation to other cities in Brazil and other countries. Thus, the degree of generalization of the results for other regions is not yet known; c) the analysis carried out with children aged from nine to eleven years limits the extrapolation of the results to younger or older children; d) seasonal factors, such as rainfall and temperature, were not controlled.

This study shows that, in boys, steps/day and MVPA are negatively associated with BMI, regardless of race, annual family income, education of parents, screen time, diet pattern, and sedentary time.

The cutoff point values identified are useful in the monitoring and prescription of steps/day and MVPA in boys, aiming at achieving adequate levels of BMI. At least 10,502 steps/day or 66.70 min/day of MVPA are suggested for boys, in order to maintain an appropriate BMI. In girls, at least 8,540 steps/day or 46.59 min/day of MVPA are suggested. However, this value should be viewed with caution, since, in girls, the study showed low discriminatory power.

The walking of 9,703 steps/day for boys and 9,445 steps/day for girls ensures that the children will achieve the recommendation of MVPA of 60 min/day. We suggest, for steps/day and MVPA, studies with different ages and populations, with different designs, so as to inform the cause and effect relationship with various health parameters.
